# Investigation of Factors Affecting Shuttle Walking Performance at Increased Speed for Patients with Chronic Obstructive Pulmonary Disease

**DOI:** 10.3390/jcm12144752

**Published:** 2023-07-18

**Authors:** Rukiye Çiftçi, Ahmet Kurtoğlu, Özgür Eken, Dilber Durmaz, Serdar Eler, Nebahat Eler, Monira I. Aldhahi

**Affiliations:** 1 Department of Anatomy, Medical Faculty, Gaziantep Islamic Science and Technology University, Gaziantep 27260, Turkey; rukiyekelesciftci@hotmail.com; 2 Department of Coaching Education, Sport Science Faculty, Bandirma Onyedi Eylul University, Balikesir 10250, Turkey; kurtogluahmet18@gmail.com; 3 Department of Physical Education and Sport Teaching, Inonu University, Malatya 44000, Turkey; ozgureken86@gmail.com; 4 Department of Thoracic Diseases, Balikesir, Medical Faculty, Bandirma Onyedi Eylul University, Bandırma 10250, Turkey; dilberdurmaz@bandirma.edu.tr; 5 Department of Coaching Education, Faculty of Sport Sciences, Gazi University, Ankara 06560, Turkey; seler@gazi.edu.tr; 6 Department of Coaching Education, School of Physical Education and Sports, Zonguldak Bulent Ecevit University, Zonguldak 67100, Turkey; nebahateler@beun.edu.tr; 7 Department of Rehabilitation Sciences, College of Health and Rehabilitation Sciences, Princess Nourah bint Abdulrahman University, P.O. Box 84428, Riyadh 11671, Saudi Arabia

**Keywords:** Medical Research Council scale, chronic obstructive pulmonary disease, respiratory function, shuttle test, COPD, dyspnea

## Abstract

The aim of this study was to examine the factors affecting the shuttle walking test (SWT) in patients with chronic obstructive pulmonary disease (COPD). A total of 29 patients with COPD (the COPD group) and a healthy group (HG) of 34 women aged between 55 and 74 years were included in the study. After the pulmonary function profiles of the participants were assessed, and the SWT was performed. Walking distances, walking speeds, and SWT levels (SWT-L) were determined with the SWT. Before and after the SWT, the heart rate (HR), oxygen saturation level (SPO_2_), and Borg scale (perceived exertion (BSe) and dyspnea (BSd)) results were analyzed with a paired sample *t*-test. The dyspnea levels during activity of daily living were determined with the Medical Research Council (MRC) dyspnea scale, and the relationship between MRC dyspnea (MRCD) and walking distance, speed, and SWT-L was tested using multiple linear regression and Pearson correlation analysis. The walking distance, speed, and SWT-L were lower in the COPD group (*p* < 0.001) than in the HG. The HR values before and after the SWT changed significantly in the COPD group and the HG (*p*< 0.001), and the effect size was higher in the COPD group. Although the BSe and BSd results before and after the SWT in the COPD group increased significantly (*p* < 0.001), they did not change in the HG. There was a highly negative correlation between MRCD and walking distance, speed, and SWT-L in the COPD group (*p* = 0.002, *p* = 0.000, and *p* = 0.001, respectively), but no correlation was found in the HG. The results showed that the HR, perceived exertion, and dyspnea levels of women with COPD whose respiratory functions were lower than the HG were significantly affected on the SWT.

## 1. Introduction

The ability to perform daily physical activities requires the integration of multiple systems, including the cardiovascular, pulmonary, and autonomic nervous systems, and the respiratory/peripheral muscles, which are inexorably linked to the integrity of exercise performance [1]. In patients with chronic obstructive pulmonary disease (COPD), heterogeneity in clinical presentation and disease progression is evident in the clinical setting [2]. Nearly 600 million individuals worldwide are affected by COPD, which is recognized as one of the major causes of chronic morbidity and mortality [3]. COPD is distinguished by airflow limitation, which is usually irreversible [4]. The progression of the disease results in the deterioration of lung function and an increased risk of hypoxemia [5]. In COPD conditions, increased airway resistance or decreased lung compliance can lead to heightened work of breathing [6]. A growing body of evidence has indicated that dyspnea can arise from various causes in patients with COPD, including increased work of breathing [7]. The increased effort required for adequate respiration can result in dyspnea, as patients perceive the sensation of breathlessness. Dyspnea and increased work of breathing may influence an individual’s ability to tolerate exercise and perform physical activity efficiently.

Dyspnea is a key symptom of COPD and can influence the perception of exertion and sustained physical activity [8]. Studies have suggested that the mechanism for dyspnea in patients with COPD is complex and may involve alterations in respiratory muscle function, neurophysiological pathways, and the sensory and affective processing of information related to breathing. Several mechanisms contribute to the sensation of dyspnea [9]. Peripheral chemoreceptors in the carotid bodies and central chemoreceptors in the brainstem respond to changes in arterial oxygen and carbon dioxide levels, triggering the perception of dyspnea [9]. Additionally, afferent signals from mechanoreceptors in the respiratory muscles and lungs, as well as neural inputs from higher centers in the brain, play a role in the perception of dyspnea [9]. Traditionally, the respiratory system has not been considered a limiting factor in exercise performance. However, many studies in the broader literature have demonstrated that following high-intensity whole-body exercise or prolonged submaximal exercise, respiratory muscle fatigue tends to develop [10]. Restricted muscle oxygenation and blood flow are major contributors to physical performance impairment and fatigue in patients with COPD [11]. In this respect, scientific evidence has shown that the inability of the respiratory muscle to sustain exercise-induced hyperpnea and high metabolic demand influences exercise performance [12,13]. The effect of increased breathing and dyspnea on exercise workload, such as speed and distance, must be investigated in detail among patients with COPD. Walking speed reflects overall well-being, captures the multisystemic effects of disease severity, is associated with risk of disability [14] and hospitalization, and can be used to predict survival [15]. However, whether walking speed and distance affect the severity of dyspnea in a dynamic context is unknown.

Individuals with COPD experience difficulty executing functional activity because of symptoms such as fatigue and shortness of breath [16]. Walking speed could be a potential factor that impacts dyspnea. Several scientific mechanisms elucidate that slow walking requires the maintenance of a stable gait pattern, which involves a prolonged stance phase and decreased stride length. This altered gait pattern leads to increased energy expenditure, as more muscle contractions are required to sustain forward movement at a reduced pace [17,18]. Consequently, the metabolic demand for slow walking is higher than that for normal or faster walking speeds, resulting in elevated energy expenditure. A slow gait speed requires greater muscular effort to generate and control movement, resulting in reduced mechanical efficiency. Understanding the relationship between walking speed and dyspnea can aid in evaluating functional limitations, designing targeted interventions, and improving the management of individuals experiencing dyspnea during slow walking.

The incremental SWT was developed to assess the maximum exercise capacity at which a person walks at an increasing pace until they reach a symptom-limited peak performance [19]. This test is a simple, inexpensive, and clinically relevant screening tool for evaluating patients with COPD for exercise capacity [20,21]. Patients with decreased physical performance and increased perception of dyspnea tend to avoid activities of daily living. This vicious cycle leads to musculoskeletal disorders. In recent years, studies have been conducted to clarify the relationship between COPD and musculoskeletal problems [22,23].

Patients with COPD often experience dyspnea and exercise-induced breathlessness. Dyspnea can affect the walking performance of patients with COPD and may subsequently lead to reduced physical activity and poor quality of life. However, the relationship between walking speed and distance and dyspnea in patients with COPD is not well understood and requires further investigation. Therefore, the aim of this study was to assess the pulmonary function, walking performance, and physical exertion parameters, such as the heart rate (HR), Borg scale, and dyspnea level of individuals with COPD, and to test whether there is a relationship between dyspnea and walking speed and distance for individuals with COPD. In this context, we hypothesized that walking speed and distance predict the severity of dyspnea among patients with COPD.

## 2. Materials and Methods

### 2.1. Study Design and Participants

In this cross-sectional study, G-power software 3.1.9.7. (University of Duesseldorf, Germany) was used to calculate the minimum number of required samples. The program utilized mean values and a priori calculations based on the given α, power, and effect size. With an α error sample of 0.05, a minimum effect size of 0.50, and a power of 0.80, the minimum number of participants was calculated to be 51 to achieve an actual power of 80.5%. As a result, a total of 63 women aged 55–74 years, who met the inclusion criteria, participated. Of the participants, 29 were women diagnosed with COPD (the COPD group) and 34 were healthy women (the healthy group (HG)). The COPD group was classified according to GOLD criteria. Accordingly, those with FEV1/FVC ratios below 70% were included in the COPD group. Then, the participants were evaluated according to FEV1: 4 participants had very severe COPD (FEV1 < 30%), 20 participants had severe COPD (30% < FEV1 > 50%), and 5 participants had mild COPD (50% < FEV1 < 80%) [24]. A convenient sampling method was used in which the inclusion criteria for the COPD group were that they were literate, could walk, did not have cognitive problems, had not undergone thoracic surgery, and were between the ages of 55 and 74. The inclusion criteria for the control group were literate, were between the ages of 40 and 70, had not been diagnosed with COPD, and did not have any lung, heart, or orthopedic disorders. Individuals with chronic asthma, significant musculoskeletal problems, acute infections, metabolic and cardiovascular diseases, and respiratory diseases other than COPD, or who used beta-blockers, were excluded from the study.

### 2.2. Study Procedure

Participants were informed that they should avoid physical exertion before testing and sleep at least eight hours a night, avoid drinking food or beverages other than water until at least three hours before the test, and avoid caffeine-like beverages. Clinical tests and functional tests were performed between 13:00 and 14:00, so that circadian rhythms and chronotypes affected the test results. HR, oxygen saturation (SPO_2_), dyspnea level, and Borg scale measurements were conducted before and after the SWT. In addition, the SWT level and walking speed and distance were determined. The Medical Research Council (MRC) dyspnea scale was used to determine the participants’ dyspnea levels.

### 2.3. Measurement

#### 2.3.1. Anthropometric Measurement

The patients were instructed to stand barefoot while having the precision of a steel stadiometer (0.1 cm) used to measure their heights in centimeters and ages in years. They were weighed using a Tanita Body Analysis System (Tanita Corporation, Tokyo, Japan), while they were metal-free and without shoes. BMIs were calculated using the following formula: weight (kg)/height (m^2^) [25].

#### 2.3.2. Heart Rate and SPO_2_ Level Measurements

Oxygen saturation and heart rate were measured using a pulse oximeter device (Spirolab III, Medical International Research) prior to and following the shuttle walking test. A quick and accurate way to evaluate oxygenation is via pulse oximetry. It is a quick, painless, safe, and effective replacement for blood collection. Due to these benefits, the oximeter is a crucial instrument for measuring an individual’s oxygen requirements and gauging the success of the given treatment. Above 95% is considered the normal oxygen saturation level, while readings below 93% need closer monitoring of the patient and the necessity for oxygen therapy [26].

#### 2.3.3. Shuttle Walk Test

The SWT is a corridor test in which the walking speed gradually increases. The test is based on the principle of increasing the walking speed per minute with acoustic stimuli and is used to determine maximum oxygen consumption. The test is conducted by measuring the patient’s ability to walk a certain number of meters or laps during a round trip between two points that are 10 m apart. This methodology, which is gaining popularity, provides valuable insight into the patient’s physical fitness. The walking speed starts at 0.5 m/s and increases by 0.17 m every second. There are 12 levels. The completion criterion for the test is the observation of fatigue or symptoms. The SWT is useful for determining maximum oxygen consumption (MaxVO_2_), and it is ideally suited for evaluating patients after pulmonary rehabilitation programs [27,28].

#### 2.3.4. Pulmonary Function Test

The physiological test known as a pulmonary function test (PFT) numerically measures a person’s lung functioning. A portable spirometer (Spirolab, SDI Diagnostics, South Easton, MA, USA) was used for the testing. The experiments were carried out at 25 °C or room temperature. Peak expiratory flow (PEF), forced expiratory flow (FEF25-75) at 25% to 75% of vital capacity, and forced expiratory volume in one second (FEV1) were all recorded. Another spirometry technique was used to measure maximum voluntary ventilation. All participants were subject to acceptance standards [29].

#### 2.3.5. Medical Research Council (MRC) Dyspnea Scale

In this study, participants’ perceived shortness of breath (dyspnea) was determined by the MRC dyspnea questionnaire [30]. The patients provided self-reported assessments of their degree of breathlessness and were subsequently stratified into MRC dyspnea grades 3, 4, or 5 based on the severity of their perceived functional impairment. The items on the MRC scale were scored between 0 and 5, with 0 representing dyspnea with vigorous exercise and 5 representing breathlessness to get dressed or leave the house [31].

#### 2.3.6. Modified Borg Scale

In the present study, the Borg scale (perceived exertion (BSe)) and the Borg dyspnea scale (BSd) were used. Although the Borg scale is frequently employed today to define the severity of exertional dyspnea, the scale can also be used to evaluate the severity of resting dyspnea [32]. The BSd was scored 0–10 (0 = no dyspnea, 3 = mild, 5 = severe, 8 = very severe, and 10 = most severe dyspnea). Similarly, the BSe was scored between 0 and 10 (0 = no exertion, 0.5 = barely noticeable, 1 = very light, 2 = light, 3 = moderate, 4 = somewhat hard, 5 = hard, 7 = moderately hard, 9 = very hard, and 10 = extremely hard exertion) [33].

### 2.4. Statistical Analszes

Statistical analyses were performed with SPSS package program 25. To assess the data, normality analyses were tested with the Kolmogorov–Smirnov test. In addition, Levene’s test was applied for homogeneity of variances. The results were reported as the mean ± standard deviation, accompanied by the 95% confidence interval (CI), as the data were found to be normally distributed. Therefore, an independent-sample *t*-test was performed to compare the respiratory function of the COPD group and the HG and the walking distance and speed and SWT-L results. In addition, a paired-sample *t*-test was applied to compare the HR, SPO_2_, BSe, and BSd results before and after the SWT. A multiple linear regression analysis was performed to determine the relationship between the MRC dyspnea scale and walking distance and the speed and SWT-L. This method assesses the linear association between the independent variables (walking speed and distance, level) and the dependent variable (dyspnea). In the study, effect sizes (ESs) were calculated and classified according to Cohen’s d formula to determine the magnitude of the differences between the experimental conditions. An ES of 0.2 was classified as small, 0.5 as medium, and 0.8 as large [34]. In this study, a *p* value of less than 0.05 was considered statistically significant.

## 3. Results

Table 1 displays the statistical analyses of the demographic information of participants. The demographic characteristics of the COPD group (*n* = 29), including age and BMI, did not differ significantly from those of the HG (*n* = 34) (*p* > 0.05). However, there were significant differences in height between the COPD group and the HG (*p* = 0.006). This indicates that patients with COPD tend to be taller. Regarding the pulmonary function parameters, significant differences were observed between the COPD group and the HG across all measured parameters (*p* < 0.001).

The pulmonary function test in the COPD group showed that forced vital capacity (FVC) ((ES)= 1.42, *p* < 0.001), FVC (%) (ES = 1.84, *p* < 0.001), FEV1 (ES = 2.62, *p* < 0.001), FEV1% (ES = 3.11, *p* < 0.001), FEV1/FVC (ES = 3.80, *p* < 0.001), PEF (ES = 2.16, *p* < 0.001), and PEF% (ES = 3.14, *p* < 0.001). These findings indicate impaired pulmonary function in individuals with COPD. 

The distance covered during the SWT was significantly lower in the COPD group (208.62 ± 89.67 m) than in the HG (333.82 ± 56.67 m). The ES, measured with Cohen’s d, was 1.66, which is large. In addition, the walking speed during the SWT was also significantly slower in the COPD group by 1.04 m/s (Table 2).

The participants’ HR, SPO_2_, and Borg scale ratings before and after the SWT are presented in Table 3. HR increased significantly from the pretest to the posttest in the COPD group and the HG (*p* < 0.001). The SPO_2_ levels remained relatively stable in the COPD group. In contrast, the HG experienced a significant decrease of 3.18% in SPO_2_ levels within the normal range. The BSe and BSd ratings increased significantly from the pretest to the posttest in the COPD group compared to the HG, which indicated significant changes in exertion and the perception of dyspnea following the SWT.

In the COPD group, there was a high correlation between the MRC dyspnea and the SWT result (*p* = 0.002, r = −0.540, 95% CI [−0.007, −0.002]), walking speed (*p* = 0.000, r = −0.609, 95% CI (−0.856, −0.275)), SWT-L (*p* = 0.001, r = −0.601, and 95% CI (−0.228, −0.071)). There was no correlation between the MRCD scale grade and the walking distance and speed and SWT-L results in the HG (*p* > 0.05). The multiple linear regression results showed that walking distance and speed and SWT-L tend to explain the variation in dyspnea during physical activity by 45. The regression analysis revealed that walking distance, walking speed, and SWT level were significant predictors of the MRCD scale in the COPD group. These results suggest that short walking distance, slow walking speed, and low SWT-L are associated with higher perceived dyspnea levels (Table 4).

## 4. Discussion

The demonstration of walking capacity through field tests has become popular in recent years and provides tools for measuring aerobic performance that can be performed outside the laboratory setting [35]. We aimed to characterize the physical performance pulmonary function profile and HR response to walking and to investigate the predictive effect of walking speed on the perception of dyspnea. The results showed that the walking distances, speed, and SWT-L were higher in the HG than in the COPD group. Furthermore, compared to healthy controls, dyspnea and exertion after shuttle walking were significantly higher in patients with COPD. In the COPD group, the pre-posttest comparison showed that the HR, BSf, and BSd results after the SWT were adversely affected. In addition, there was a significant negative correlation between MRC dyspnea scale and walking distance, speed, and SWT-L in the COPD group. To the best of our knowledge, the present study is the first to examine the factors affecting the walking mechanism in patients with COPD.

Functional performance was low in patients with COPD, which is in accordance with a previous study. Donaire-Gonzalez et al. [36] reported that daily activities were performed for shorter durations and at lower intensities by patients with severe and very severe COPD compared to individuals with mild and moderate stages of COPD. Different rehabilitation processes are known to positively affect the pulmonary functions of patients with COPD. In a study, Croitoru et al. [37] found that a 7-week pulmonary rehabilitation program for patients with COPD led to an improvement in physical performance. In this study, walking speed was lower in patients with COPD than in the HG. In the regression analysis, we found that the MRC dyspnea scale result was associated with walking speed in patients with COPD. According to this result, a high perception of dyspnea was associated with walking speed and distance.

Pulmonary function test parameters, which are evaluated as objective findings in addition to clinical signs and symptoms, are important in the diagnosis of patients with COPD [4]. In this study, it is evident that patients with COPD showed a decline in walking performance and distance, which aligns with a previous study that highlighted a decrease in cardiopulmonary capacity and fitness [38]. A potential factor that might moderate this decline in functional performance is lung diffusion capacity. A study found that the low diffusion capacity of the lung for carbon monoxide, particularly measured using the single-breath method, can suggest a more emphysematous pathophysiology and has been associated with worse exercise tolerance and exercise-induced oxygen desaturation [38]. Peripheral factors also play a role in exercise intolerance in patients with COPD. Muscle atrophy, weakness, and fatigue are common in patients with COPD, leading to decreased muscle function and endurance [39,40]. Peripheral muscle dysfunction in COPD is characterized by oxidative stress, impaired muscle metabolism, and reduced muscle mass [40]. These factors contribute to decreased exercise capacity and tolerance. Furthermore, psychological factors, such as anxiety and depression, have been reported to influence walking performance [41]. In addition, the relationship between skeletal muscle oxygenation and exercise has been investigated. It has been reported that peripheral skeletal muscle oxygenation is not compromised in patients with COPD during submaximal exercise [42]. This suggests that limitations in exercise capacity in patients with COPD are more likely a result of muscle disuse and mitochondrial dysfunction [43] and poor lung function, rather than impaired muscle oxygenation. It has been reported that there is a relationship between skeletal muscle oxygenation and systemic oxygen uptake during exercise in patients with COPD, which is similar to that found in healthy subjects. The study found that muscle oxygenation was negatively correlated with systemic oxygen uptake and positively correlated with the HR and the muscle oxygen extraction rate [44]. The relationship between skeletal muscle oxygenation and exercise in COPD is complex and can be influenced by various factors, such as lung function, cardiovascular adjustments, and interventions.

Exercise testing is frequently used to evaluate a patient’s COPD-related impairment and treatment response. The 6-minute walk test (6MWT) and the SWT have been employed in the literature to evaluate pulmonary function, health-related quality of life, maximum exercise capacity, and mortality in patients with COPD [45]. In this study, we used the SWT to assess the functional status of the COPD group and the HG. According to the test results, the walking speed and level were higher in the HG. These results show that walking function slows in patients with COPD. Based on the strong relationship between measures of exercise capacity and other important outcomes in COPD, we believe that walking speed can be used as an indicator of general well-being in a clinical setting.

The measurement of dyspnea using the Borg scale before and after the SWT can assist in assessing a patient’s oxygenation levels, at rest and during exercise [46]. HR changed significantly in both groups compared to before and after the SWT. The BSe and BSd results changed only in the patients with COPD. A previous study [47] found an inverse proportion between the measurement of the perception of dyspnea through the Borg scale before the test and the walking distance. In this case, the degree of airway limitation may play an important role in the development of dyspnea after rest and exercise. The results of this study show the relationship between the MRCD scale and the SWT in patients with COPD.

The MRC dyspnea scale is an important preliminary test for planning the rehabilitation process of individuals with COPD. Individuals with COPD with an MRCD grade of 3 or higher have been shown to have a clear exercise intolerance, decreased general health, decreased mood, and a decrease in self-reported daily physical activity [48]. In this study, we observed that when the MRCD increased, the walking distance and speed and SWT-L values decreased in parallel with the literature results. In this case, we think that the MRCD scale is important for predicting the results of certain functional tests, especially for individuals with COPD.

The main methods used in the evaluation of dyspnea in patients with COPD are the MRC dyspnea scale and the modified Borg scale. The MRCD scale passively examines the patient, but the modified Borg scale examines the patient during exercise using pulse oximetry. It has been reported that the modified Borg scale is essential for confirming MRCD values [49]. In this study, the pretest and posttest scores on the modified Borg scale differed significantly in the COPD group. There was no difference in scores in the HG. These results indicate that all factors affecting fatigue in patients with COPD should be assessed. Thus, walking speed and distance are negatively affected in patients’ dyspnea during activity, as measured with the MRC scale [50]. The regression analysis revealed that walking distance, walking speed, and SWT level were significant predictors of MRCD scores in the COPD group. These findings suggest that longer walking distance, faster walking speed, and higher SWT-L scores are associated with lower perceived dyspnea levels. However, it is important to note that the model accounted for a small amount of the variance in the MRCD scores, indicating that other factors not included in the model may also contribute to perceived dyspnea among the study respondents.

The present study has several limitations that should be considered when interpreting the findings. First, the sampling method employed in this study included only female participants. The generalizability of the study findings to male individuals is limited because of the insufficient voluntary participation of men in the research. Consequently, it is necessary to conduct comprehensive investigations involving male participants to address this gap in knowledge. Second, the measurement of dyspnea and exertion relied on subjective, self-reported measures. Although widely used and accepted in research and clinical practice, self-reported measures are susceptible to individual interpretation and recall biases. Objective measures, such as physiological monitoring or standardized scales, could provide more precise and reliable data for assessing dyspnea and exertion levels. Incorporating objective measures into future studies would enhance the accuracy and objectivity of these assessments. Thus, this study utilized dyspnea and heart rate as exercise intolerance markers, and while informative, more accurate results could be obtained with the inclusion of additional biochemical markers such as lactic acid, cytokines, or oxidative parameters. Third, the observed difference in height between participant groups constitutes a significant limitation of the study. Height is an essential determinant of lung volume and pulmonary function, potentially influencing respiratory parameters and exercise performance. Fourth, the assessment of functional capacity in this study was conducted using a field test, namely the SWT. Field tests have advantages in terms of simplicity and ease of administration and have previously undergone validation and demonstrated reliability as a suitable tool for evaluating functional capacity. The testing may not fully capture the complexities of overall functional capacity. Employing a combination of field tests and laboratory-based assessments, such as cardiopulmonary exercise testing, would provide a more comprehensive evaluation of functional capacity, including cardiovascular and pulmonary parameters. As a limitation of this study, we acknowledge the need for further investigation in future studies to address this concern.

## 5. Conclusions

This study showed that individuals with COPD had significantly lower respiratory function, walking distance and speed, and SWT-L results. In addition, the COPD group experienced increased self-perceived exertion, dyspnea, and HR after the SWT. These findings provide insight into the factors that hinder daily routine walking performance in patients with COPD. This study showed that the MRCD scale is a crucial tool for predicting the efficacy of exercise interventions such as walking distance and speed and the SWT-L in individuals with COPD. This information is pertinent for healthcare professionals, exercise specialists, and physicians who are responsible for the care of such patients. Future research incorporating a wider range of functional movements is likely to enrich the generalizability of our findings and add to the literature.

## Figures and Tables

**Table 1:** Demographic and pulmonary function profile characteristic of the participants.

Parameters	COPD N = 29	HG N = 34	*p*-Value
	M ± SD	M ± SD	
Age (years)	63.41 ± 4.76	63.52 ± 4.74	0.924
Weight (kg)	69.78 ± 14.78	66.84 ± 8.63	0.329
Height (cm)	167.20 ± 9.72	161 ± 5.99	0.006
BMI (kg/m^2^)	24.80 ± 3.91	25.57 ± 4.30	0.462
FVC (L)	1.68 ± 0.50	2.28 ± 0.32	<0.001
FVC% predicted	52.13 ± 14.40	79.02 ± 14.79	<0.001
FEV_1_ (L)	1.07 ± 0.42	2.05 ± 0.32	<0.001
FEV_1_% predicted	41.03 ± 10.53	87.88 ± 18.45	<0.001
FEV_1_/FVC (L)	63.37 ± 7.16	89.58 ± 6.62	<0.001
PEF (L)	2.13 ± 079	4.53 ± 1.35	<0.001
PEF% predicted	32.00 ± 9.61	77.32 ± 17.98	<0.001

BMI: body mass index, COPD: chronic obstructive pulmonary disease, HG: healthy group, FVC: forced vital capacity, FVC_1_: forced expiratory volume in one second, PEF: peak expiratory flow rate.

**Table 2:** Demonstrate the functional performance of participants.

Shuttle Walking Test Parameters	COPD N = 29 M ± SD	HG N = 34 M ± SD	t-Value	Cohen’s d	*p*-Value
Distance (meter)	208.62 ± 89.67	333.82 ± 56.67	−6.494	1.66	<0.001
Speed (m/s)	3.17 ± 0.82	4.21 ± 0.37	−6.331	1.63	<0.001
SWT-Level	5.75 ± 3.05	9.47 ± 1.52	−5.944	1.54	<0.001

SWT: shuttle walking test.

**Table 3:** Comparison of participants’ HR and Borg scale results before and after shuttle walking test.

Parameters	Group	Pretest	Posttest	t-Value	*p*-Value
HR (b/min)	COPD	82.34 ± 16.20	106.10 ± 18.14	−7.850	<0.001
	HG	78.61 ± 13.09	100.20 ± 21.25	−6.114	<0.001
SPO_2_ (%)	COPD	90.68 ± 8.47	89.17 ± 6.17	1.306	0.202
	HG	96.94 ± 1.73	93.79 ± 3.82	4.454	<0.001
BSe	COPD	1.20 ± 1.42	3.93 ± 1.98	−8.102	<0.001
	HG	0.17 ± 0.57	0.26 ± 0.75	−1.000	0.325
BSd	COPD	2.51 ± 0.82	4.89 ± 1.69	−6.168	<0.001
	HG	0.23 ± 0.65	0.35 ± 0.73	−0.643	0.524

HR: heart rate, SPO_2_: oxygen saturation, BSe: Borg scale exertion, BSd: Borg scale dyspnea, COPD: chronic obstructive pulmonary disease, HG: healthy group.

**Table 4:** Multiple linear regression models with the MRC as dependent variables among study respondents.

Dependent Variables	Predictors	Coefficients							R^2^
		B	SE	β	t	*p*-Value	95% CI		
							Lower	Upper	
**MRC**	**COPD**								
	CONSTANT	5.132	2.043		2.512	0.019 *	0.924	9.339	0.45
	Walking Distance	−0.005	0.001	−0.540	−3.338	0.002 *	−0.007	−0.002	
	Walking speed	−0.565	0.142	−0.609	−3.994	<0.001 *	−0.856	−0.275	
	SWT-L	−0.150	0.038	−0.602	−0.3917	0.001 *	−0.228	−0.071	

* Denotes statistically significant value of *p*-value ≤ 0.05; B: unstandardized beta regression coefficient; SE: standard error; β: standardized beta, SWT-L: shuttle walking test-level.

## Data Availability

The data presented in this study are available on request from the corresponding author.

## Abbreviations

BSe	Borg scale (perceived exercion)
BSd	Borg scale (perceived dyspnea)
COPD	Chronic obstructive pulmonary disease
FEV1	Forced expiratory volume in one second
FVC	Forced vital capacity
HG	Healty group
HR	Heart rate
MaxVO_2_	Maximum oxygen consumption
MRCD	Medical Research Council dyspnea
PEF	Peak expiratory flow
PFT	Pulmonary function test
SPO_2_	Oxygen saturation level
SWT	Shuttle walking test
WS	Walking speed
SWT-L	Shuttle walking test level
